# Deep Learning–Based SD-OCT Layer Segmentation Quantifies Outer Retina Changes in Patients With Biallelic RPE65 Mutations Undergoing Gene Therapy

**DOI:** 10.1167/iovs.66.1.5

**Published:** 2025-01-02

**Authors:** German Pinedo-Diaz, Birgit Lorenz, Sandrine H. Künzel, Sarah Thiele, Susana Ortega-Cisneros, Eduardo Bayro Corrochano, Frank G. Holz, Alexander Effland

**Affiliations:** 1 Center for Research and Advanced Studies, Cinvestav, Zapopan, Mexico; 2 Dept of Ophthalmology, University Hospital, Bonn, Germany; 3 Transmit Centre for Translational Ophthalmology, c/o Justus-Liebig-University Giessen, Giessen, Germany; 4 Department of Ophthalmology, University Medical Center Hamburg-Eppendorf, Hamburg, Germany; 5 Institute for Applied Mathematics, University of Bonn, Bonn, Germany

**Keywords:** *RPE65*-IRD, SD-OCT, ellipsoid zone biomarkers, gene augmentation therapy, deep learning–based image segmentation

## Abstract

**Purpose:**

To quantify outer retina structural changes and define novel biomarkers of inherited retinal degeneration associated with biallelic mutations in *RPE65* (*RPE65*-IRD) in patients before and after subretinal gene augmentation therapy with voretigene neparvovec (Luxturna).

**Methods:**

Application of advanced deep learning for automated retinal layer segmentation, specifically tailored for *RPE65*-IRD. Quantification of five novel biomarkers for the ellipsoid zone (EZ): thickness, granularity, reflectivity, and intensity. Estimation of *the* *EZ_area_* in single and volume scans was performed with optimized segmentation boundaries. The control group was age similar and without significant refractive error. Spherical equivalent refraction and ocular length were evaluated in all patients.

**Results:**

We observed significant differences in the structural analysis of EZ biomarkers in 22 patients with *RPE65*-IRD compared with 94 healthy controls. Relative EZ intensities were already reduced in pediatric eyes. Reductions of EZ local granularity and EZ thickness were only significant in adult eyes. Distances of the outer plexiform layer, external limiting membrane, and Bruch's membrane to EZ were reduced at all ages. EZ diameter and area were better preserved in pediatric eyes undergoing therapy with voretigene neparvovec and in patients with a milder phenotype.

**Conclusions:**

Automated quantitative analysis of biomarkers within EZ visualizes distinct structural differences in the outer retina of patients including treatment-related effects. The automated approach using deep learning strategies allows big data analysis for distinct forms of inherited retinal degeneration. Limitations include a small dataset and potential effects on OCT scans from myopia at least −5 diopters, the latter considered nonsignificant for outer retinal layers.

*R* *PE65* codes for an isomerohydrolase in the RPE essential for retinol recycling.^1^^–^^4^ Biallelic mutations in *RPE65* are often associated with a rare form of inherited retinal degeneration (*RPE65*-IRD) with progressive atrophy of the RPE and the photoreceptors together with severe vision impairment to blindness.^5^ Successful phase 1, 2, and 3 studies with subretinal gene augmentation therapy^6^^,^^7^ have resulted in the approval of voretigene neparvovec (VN [Luxturna]) for clinical use. Several postmarketing studies have been published, including our own consecutive series of 30 eyes from 19 patients.^8^ An ongoing challenge is achieving objective, high-resolution quantification of the natural progression of the disease and the therapeutic effect, especially in larger patient cohorts and within routine clinical settings.

The ellipsoid zone (EZ), formerly known as the inner/outer segment of photoreceptors, refers to the second hyper-reflective band in optical coherence tomography (OCT) of the retina. An intact EZ has been reported to be associated with good visual function and visual field in a variety of retinal diseases, including IRDs.^9^^–^^12^

However, assessments of the EZ have been typically qualitative in nature or limited to a single B-scan. Traditional segmentation methods often require manual intervention and have limitations in handling complex image structures with ambiguous boundaries, making manual segmentation challenging and leading to low segmentation repeatability. Manual segmentation is prone to interobserver and intraobserver variability, which can lead to inconsistencies and errors in the results.^13^

In recent years, deep learning (DL)–based segmentation methods such as convolutional neural networks, have shown promising results in segmenting medical images in IRDs and other retinal conditions such as fundus autofluorescence and SD-OCT images accurately without requiring manual intervention.^14^^–^^16^ Convolutional neural network–based segmentation methods have proven robust in handling the variability in image quality and can provide segmentation results in real time.^17^

The presented work highlights the power of SD-OCT layer segmentation using an automated DL-based approach in the context of 22 patients with *RPE65*-IRD compared with 94 healthy controls. We address the challenge of high time consumption for manual annotation and validate the results for repeatability and reproducibility. Using a modified U-Net DL architecture^18^ for precise retinal layer segmentation, we extend the segmentation to all outer retinal layers, that is, the outer plexiform layer (OPL), EZ, external limiting membrane (ELM), and Bruch's membrane (BM) in SD-OCT images. Our approach allows us to define novel biomarkers that include thickness, granularity, reflectivity, and relative intensity of EZ.

We further quantify EZ area in volume scans and EZ diameter in single scans using the segmentation boundaries. These objective measures can then be compared with the subjective results of perimetric visual field testing.

This approach enables comparative studies between *RPE65*-IRD patients and healthy controls, as well as longitudinal analysis of EZ biomarkers before and after treatment, offering insights into disease progression and treatment outcomes.

## Methods

### Dataset and Participants

The dataset used in this retrospective study comprises SD-OCT scans stored at the University Hospital Bonn (UKB) together with data on refraction and ocular length. This study adhered to the principles of the Declaration of Helsinki, received approval from the institutional ethics committee (AZ325/22), and the data were anonymized before analysis.

The study uses two datasets with distinct purposes, both containing data from the same *RPE65*-IRD and healthy participants. Dataset 1 (D1), retrospectively annotated manually by an expert to delineate boundaries of different retinal layers, consists of 321 single scans from 116 participants, including 22 individuals with *RPE65*IRD and 94 healthy controls used for training DL-based models. In contrast, dataset 2 (D2) is used for testing and longitudinal analysis, comprising 262 single scans (158 *RPE65*-IRD and 94 healthy controls) with single scans and volume scans. D1 and D2 include data from 18 treated patients at baseline and after treatment with VN, from Lorenz et al.^8^ and 4 new untreated patients with a milder phenotype. We included both bilateral (26 eyes) and unilateral (11 eyes) treated patients in our study, and unilateral untreated (7 eyes), totaling 44 eyes (right and left) for D2. Unlike D1, files in D2 are not annotated for retinal layer delimitation.

The participants in D1 and D2 had an average age of 22.87 ± 9.87 years, with the control group averaging 22.55 ± 7.20 years and the *RPE65*-IRD group averaging 23.52 ± 8.90 years. Overall, there were 68 males and 48 females, with 54 males and 40 females in the control group, and 22 patients, of which 14 were males and 8 were females, in the *RPE65*-IRD group. Ocular axial length measured with an IOL master (Zeiss, Oberkochen, Germany) was available in 18 treated patients. The mean ocular length was 23.42 ± 1.22 mm. Refraction was available in all patients. The mean spherical refraction (SER) was −1.11 ± 3.99. High myopia (at least −5 diopters SER) was present in 9 of these 44 eyes (20.4%).

For further details on D2, readers are referred to the Supplementary Material.

#### SD-OCT

SD-OCT scans were obtained using the Spectralis HRA-OCT2 Heidelberg Engineering (Heidelberg, Germany). A single horizontal line B-scan traversing the fovea was captured in both high-speed (768 pixels) and high-resolution (1024 pixels) settings. These scans were divided into 496 × 128 patches with a 64-pixel overlap, serving as batched input for our DL-based model. Following Lorenz et al.,^19^ SD-OCT 30° × 25° scans were acquired using a Spectralis HRA-OCT2 with follow-up mode (121 B-scans, ART 25). For cases of nystagmus or unsteady fixation, single SD-OCT line scans were registered. The B-scans were taken on different visit dates.

#### Dataset Preprocessing

D1 was divided into training and validation sets by patient using an 80/20 random split ratio. This resulted in 93 participants (75 controls and 18 patients) in the training set and 23 participants (19 controls and 4 patients) in the validation set (a total of 116 participants). This approach ensured no overlap between sets, maintaining the generalization capability of the DL-based model and avoiding data leakage.

**Figure 1.**
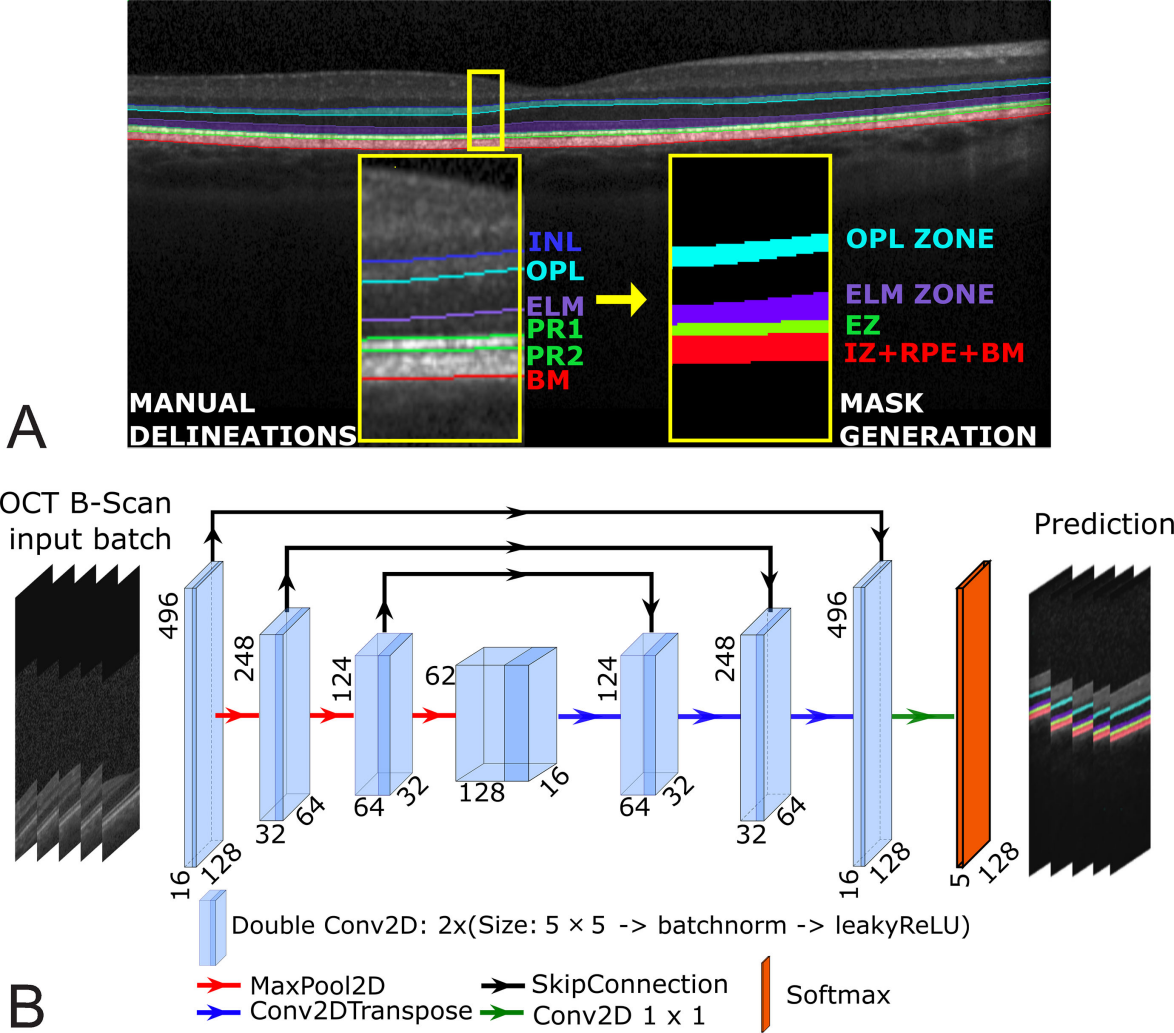
Automated B-scan layer segmentation methodology. (**A**) Annotations include distinct outer layers: the OPL zone, ELM zone, EZ, and BM. The class map mask is labeled as Background (0), EZ (1), OPL zone (2), ELM zone (3), and IZ + RPE + BM (4). (**B**) Overview of the modified four-level UNet architecture with 1.2 million parameters.

B-scans were divided into 496 × 128 patches with a 64-pixel overlap between adjacent patches. This preprocessing step was necessary to homogenize the input size for our DL-based model. Each section served as a batched input, allowing the model to process localized features across the entire scan. Moreover, each file contains a B-scan centered at the fovea, annotated by a clinical expert. An example delimitation is shown in Fig. 1A and is used to generate a mask as ground truth. Table 1 provides a comprehensive summary of the specifications and distributions within the UKB dataset.

**Table 1.** UKB Dataset Description

Parameter	All	Controls	RPE65-IRD
Participants	116	94	22
Age (average ± SD)	22.87 ± 9.87	22.55 ± 7.2	23.52 ± 8.9
SER average (SPH [D], CYL, AXIS)	—	—	−0.96, −1.97, 70.30
Ocular axial length (mm) average (R/L)	—	—	(23.37/23.39)
Sex (M/F)	(68/48)	(54/40)	(14/8)
No. of examinations of eyes (R/L)	(75/63)	(53/41)	(22/22)
SD-OCT scans in D1	321	94	227
SD-OCT scans in D2	262	94	158
Slices of D1 (train/validation)	(3572/1266)	(970/434)	(2602/832)

CYL, cylinder; L, left eye; R, right eye; SPH, sphere.

### DL Approach

For retinal layer segmentation, we use a DL-based approach using a modified U-Net architecture,^18^ which is known for its effectiveness in semantic segmentation tasks. The U-Net architecture consists of an encoder–decoder network with skip connections designed to retain both local and global context information. Further information regarding data and training is provided in the Supplementary Material.

Our modified four-level U-Net architecture, as depicted in Figure 1B, includes the following components: four-level double convolutional blocks in the encoder phase, starting with 16 filters, a kernel size of 5 × 5, and incorporating batch normalization with leaky ReLU activation. The encoder uses max pooling for downsampling. In the decoder phase, there are four levels of upsampling blocks using transposed convolutions that mirror the structure of the encoder. The architecture concludes with a final convolutional layer using five 1 × 1 filters to produce the predicted segmentation mask.

The input SD-OCT B-scan underwent partitioning into batches of 496× 128 pixel sections, resulting in an output segmentation map sized 8 × 496 ×128 ×5 per scan.

The loss function used for segmentation accuracy combines weighted cross-entropy and dice loss:

(1) $$\begin{array}{c} L_{DCE}\left(y,\hat{p}\right)=\left(1-\lambda \right)L_{Dice}\left(y,\hat{p}\right)+\lambda L_{CE}\left(y,\hat{p}\right),\; \end{array}$$

where *y* and $\hat{p}$ denote ground truth and predicted probabilities, respectively, with λ ∈ (0, 1) as a hyperparameter.

Data augmentation techniques were applied to enhance model robustness and generalization. Intensity transforms were applied to SD-OCT B-scans, and geometric adjustments were used for both images and segmentation masks (details in the Supplementary Material).

### Evaluating Retinal Changes in *RPE65*-IRD

Our focus centers on crucial and novel biomarkers primarily designed for the EZ, encompassing thickness, granularity, reflectivity, intensity, and volume area. Performing DL-based segmentation, we isolate individual layers within a 6-mm diameter region of interest, according to Early Treatment Diabetic Retinopathy Study specifications. Subsequently, we compute intensity profiles and quantify these biomarkers for both *RPE65*-IRD patients and healthy controls. Table 2 shows the summary of definitions of these biomarkers.

**Table 2.** Summary of Definitions of Biomarkers

Measure	Definition
OPL_EZ(m)	Distance between maximum EZ and OPL intensity
ELM_EZ(m)	Distance between maximum ELM intensity and maximum EZ intensity
BM_EZ(m)	Distance BM boundary and maximum EZ intensity
ELM_BM(m)	Distance between maximum ELM intensity and BM boundary
EZ_Th(m)	Thickness measurement for the EZ
*rEZI*	Intensity of EZ relative to a reference
*EZ_TV_*	Sharpness measure of the EZ

#### Weighted Peak Distances

Relative EZ reflectivity represents a pivotal biomarker used in appraising photoreceptor health.^20^^–^^23^ The relative EZ reflectivity measures the pixel distance between the peaks of the intensity profiles of EZ and OPL, offering valuable information on retinal optical characteristics and their implications for retinal diseases. Based on this approach, we introduce four novel biomarkers called weighted peak distances of the respective layers. These biomarkers measure distances between the ELM and OPL peaks, ELM and EZ peaks, BM boundary and EZ peaks, and ELM boundary and BM along the *x* axis. For evaluation, we first segment the retinal layers and extract peak values from each A-scan. Distances are then computed along the *x* axis for the B-scan centered on the fovea. Finally, the arithmetic mean and SD of these distances are calculated along the foveal B-scan. In summary, the four novel biomarkers (rescaled to micrometers) read as:

(2) $$\begin{array}{c} \;\begin{array}{c} OPL\_EZ_{i}\left(m\right)=\left|P_{max}\left(EZ_{i}\right)-P_{max}\left(OPL_{i}\right)\right|\cdot S_{y}\\ ELM\_EZ_{i}\left(m\right)=\left|P_{max}\left(ELM_{i}\right)-P_{max}\left(EZ_{i}\right)\right|\cdot S_{y}\\ BM\_EZ_{i}\left(m\right)=\left|BM_{i}-P_{max}\left(EZ_{i}\right)\right|\cdot S_{y}\\ ELM\_BM_{i}\left(m\right)=\left|P_{max}\left(ELM_{i}\right)-BM_{i}\right|\cdot S_{y}\;, \end{array}\; \end{array}$$

where $P_{max}\left(EZ_{i}\right)$, $P_{max}\left(ELM_{i}\right)$, and $P_{max}\left(OPL_{i}\right)$ denote the maximum peaks of EZ, ELM, and OPL, respectively, and *BM_i_* is the location BM boundary on the *x* axis. $S_{y}$ represents the axial resolution of the A-scan SD-OCT system (approximately 3*.*5 µm per pixel, according to the Spectralis operations manual). This process is implemented using Python, with PyTorch for segmentation, NumPy for peak value extraction and distance calculations, and iteration over the B-scan to compute the mean and SD.

**Figure 2.**
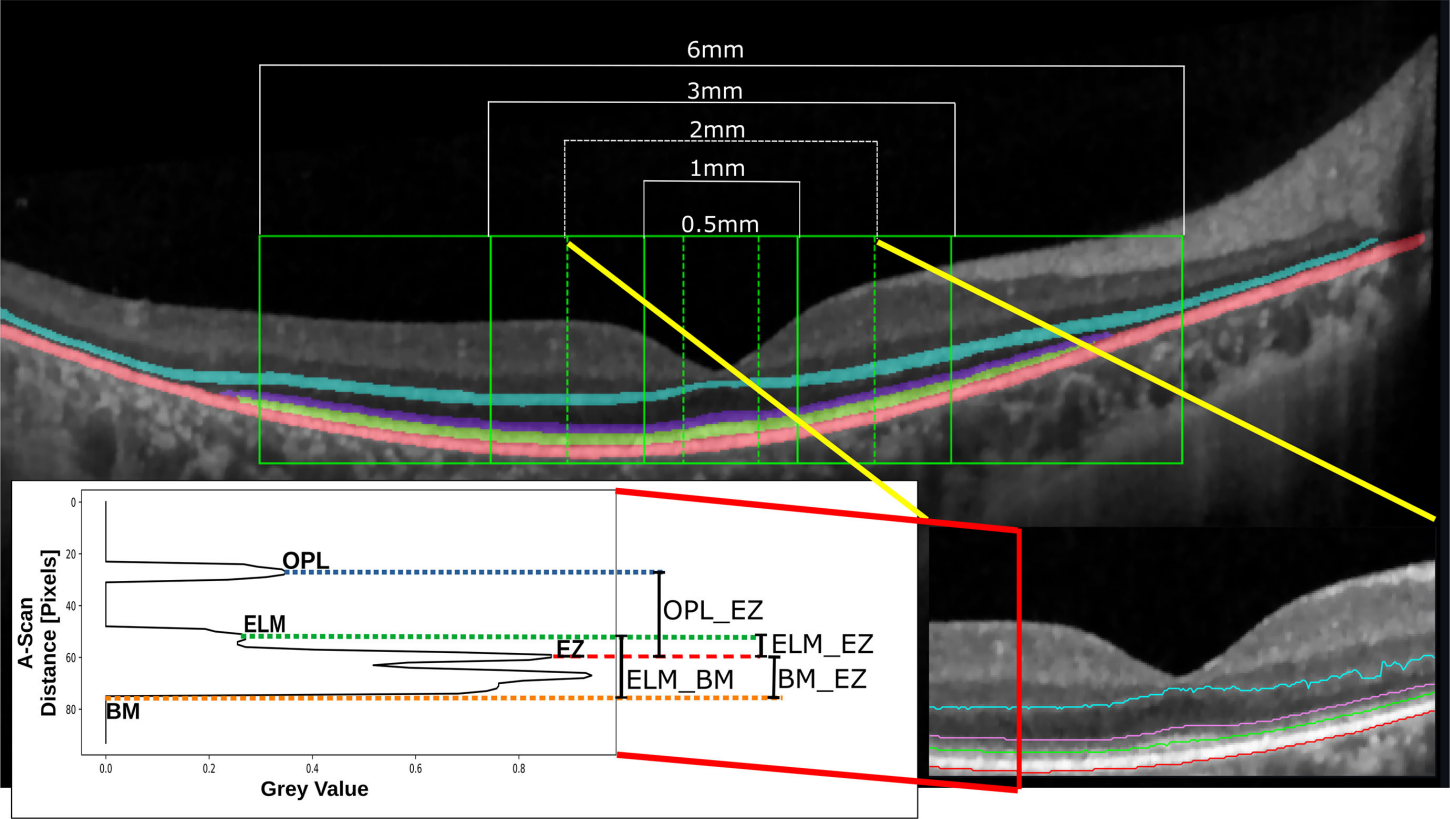
Patient P04’s right eye underwent a macular B-scan with 1536 A-scans, and baseline BCVA was 0.5 logMAR (Lorenz et al.^19^). Figure 1 shows automated segmentation results along the Early Treatment Diabetic Retinopathy Study grid (6 mm diameter). Within this region, comprising 529 A-scans, individual layer segmentation was performed. For example, intensity profiles extracted from a 2-mm segment were used to derive biomarkers like EZ reflectivity, thickness, and relative intensity, with BM localization at the boundary.

The arithmetic mean and SD of the weighted peak distances are calculated along the B-scan *x* axis, resulting in average values for OPL_EZ, ELM_EZ, BM_EZ, and ELM_BM. These biomarkers are displayed in Figure 2, segmented by the Early Treatment Diabetic Retinopathy Study grid.

#### EZ Thickness

In *RPE65*-IRD, EZ thickness seems to be different from healthy controls, which might therefore be a reliable biomarker for disease progression. We quantify EZ thickness by considering the lower and upper boundaries of the segmented EZ layer, that is:

$$\begin{array}{c} EZ_{Th}\left(m\right)=\frac{1}{N}\sum_{x=0}^{N}\left|L_{u}\left(EZ_{x}\right)-L_{\ell }\left(EZ_{x}\right)\right|\cdot S_{y},\; \end{array}$$

where *N* refers to the number of pixels in the *x*- direction and the thickness as a difference in pixels is subsequently scaled to µ*m*. $L_{u}\left(EZ_{x}\right)$ and $L_{l}\left(EZ_{x}\right)$ are the lower and upper limits of the EZ layer, respectively. S*_y_* is the axial resolution from A-scan SD-OCT system (approximately 3*.*5 µm per pixel digital according to the operations manual).

#### rEZI

In the past, most assessments of EZ have been qualitative, based on observations of its presence, absence, or the spatial extent of the disturbance.^24^ In our study on *RPE65*-IRD, we introduce a novel measure called rEZI.

Changes in retinal diseases, such as retinitis pigmentosa and AMD, which are potentially associated with mitochondrial dysfunction, have been reported. To evaluate rEZI, we measure the ratio of EZ reflectivity to OPL at each retinal location^25^ yielding

(4) $$\begin{array}{c} \mathrm{rEZI}=\frac{1}{N}\sum_{i=0}^{N}\frac{\left|P\left(EZ_{i}\right)-P\left(OPL_{i}\right)\right|}{P\left(EZ_{i}\right)},\; \end{array}$$

where *N* denotes the number of pixels on the *x* axis, and $P(EZ_{i})$, $P(OPL_{i})$ are the maximum peaks of the EZ and OPL, respectively.

#### Total Variation Denoising and EZ Granularity

**Figure 3.**
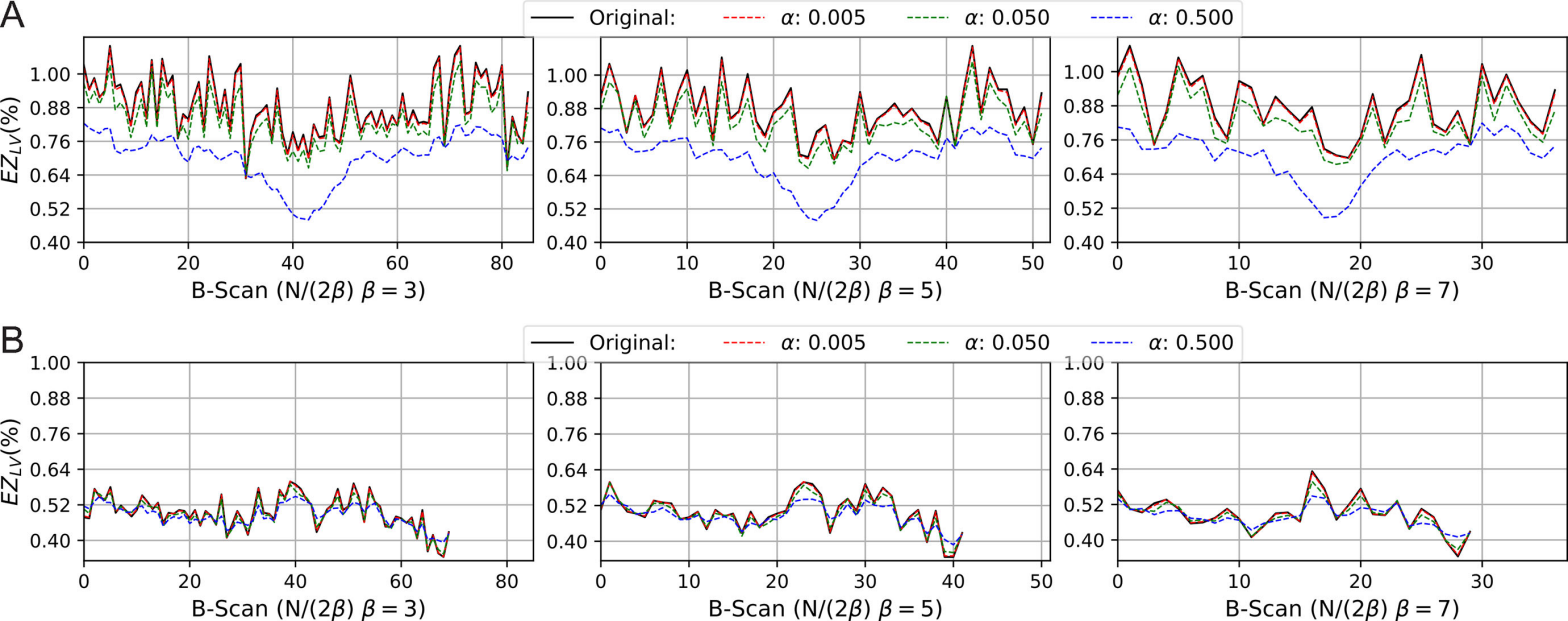
Central macular SD-OCT B-scan comparison of EZ granularity in (**A**) healthy control and (**B**) *RPE65*IRD shown in Figure 5 patient P04^19^ using TV denoising with α  ∈  {0.5,  0.05,  0.005} and β  ∈  {3,  5,  7} as explained in equations (5) and (7). The TV in (**A**) has an oscillatory behavior, with a 0.75 mean value interpreted as better EZ granularity. In contrast, (**B**) shows a constant-like behavior with 0.47 EZ granularity value. Therefore, EZ granularity between healthy and *RPE65*-IRD is significant.

EZ local granularity or EZ local variation (*EZ_LV_*) associated with *RPE65*-IRD denote the changes in the intensity of the EZ within a neighborhood, and EZ granularity or EZ total variations (*EZ_TV_*) describe its average in B scan (*x* axis). This metric measures the difference of pixel grey value in a region given by:

(5) $$\begin{array}{c} EZ_{LV}=\sum_{x=0}^{N}\sum_{k=-\beta }^{\beta }\sum_{y=0}^{M}\left|P_{x+k,y}-P_{x,y}\right|\; \end{array}$$

(6) $$\begin{array}{c} EZ_{TV}=\frac{EZ_{LV}\left(2\beta \right)}{N},\; \end{array}$$

where *N* is the total of pixels in the *x* axis, *P* is the pixel gray value, and β  ∈  {3, 5, 7} in the *x* axis is the neighborhood size. Owing to the strong dependency on noise, we additionally use a total variation-based denoising^26^ with smoothing parameter *α* in a preprocessing step. For further details, we refer the reader to the Supplementary Material. As shown in Figure 3, the local variation along B-scan (*x* axis), has been smoothed owing to TV denoising. To assess EZ granularity (*EZ_TV_*) in this study, the local variation window β = 3 and the denoising parameter α = 0.05 are established empirically, which properly removes speckle noise typically found in tomography images while promoting data consistency.

### Statistical Analysis

Biomarker comparisons were made between age groups (pediatric and adult) and healthy controls. We used the Shapiro–Wilk test to check for normality. The parametric two-sided *t* test was applied for normally distributed data (*P* *>* 0.05), and the nonparametric Wilcoxon rank-sum test was used for non-normally distributed data (*P* *<* 0.05).

Furthermore, we examined the correlation between the *rEZI* biomarker and others using Pearson's correlation coefficient. This analysis quantified the strength and direction of linear relationships between variables, with a correlation coefficient *r >* 0.3 indicating moderate to strong associations.

All statistical analyses were performed using Python 3.9 with the SciPy library for the Shapiro–Wilk test and the *t* test, and the statsmodels library for the Wilcoxon rank-sum test. Pearson's correlation was calculated using the Pandas library. The Python scripts used to perform the analyses, including image processing and DL, described herein are freely and openly available at https://github.com/alonso59/ukb_rpe65_ird/.

## Results

### Characteristics of Retinal Changes Before Treatment

Our comparative study of biomarkers extracted from SD-OCT scans at baseline diagnosed with *RPE65-*IRD and a healthy control cohort is summarized in Table 3. Data are presented as mean ± SD across three distinct comparisons: (a) comparing the control group with the pediatric patient group, (b) comparing the control group with the adult patient group, and (c) comparing the pediatric patient group with the adult patient group.

**Table 3.** Biomarkers Extracted From D2, Including *RPE65*-IRD Patients, Before Treatment and From a Healthy Control Group

	Nasal			Temporal		
Biomarker	Control	Adult	*P* Value	Control	Adult	*P* Value
Control group versus pediatric patient group						
*rEZI* (%)^*^	60.858 ± 19.68	42.389 ± 23.26	<0.0013	58.211 ± 18.16	39.997 ± 22.58	<0.0008
*EZ_TV_* (%)^†^	1.434 ± 0.31	1.395 ± 0.58	<0.7219	1.406 ± 0.32	1.293 ± 0.46	<0.2405
*EZ_Th_* (µ*m*)^†^	17.141 ± 3.06	16.267 ± 4.60	<0.3567	17.133 ± 2.34	16.149 ± 4.63	<0.2484
*OPL_EZ* (µ*m*)^†^	130.893 ± 13.76	91.920 ± 17.70	<0.0001	126.725 ± 11.20	90.757 ± 17.30	<0.0001
*ELM_EZ* (µ*m*)^*^	40.548 ± 3.97	23.020 ± 6.90	<0.0001	41.005 ± 3.56	22.925 ± 7.18	<0.0001
*BM_EZ* (µ*m*)^†^	56.894 ± 4.41	38.518 ± 6.95	<0.0001	57.898 ± 5.12	38.656 ± 6.91	<0.0001
*ELM_BM* (µ*m*)^†^	97.442 ± 7.36	61.029 ± 13.51	<0.0001	98.904 ± 7.66	59.661 ± 15.43	<0.0001
Control group versus adult patient group						
*rEZI* (%)^*^	59.434 ± 18.85	36.195 ± 20.55	<0.0001	57.541 ± 20.83	36.146 ± 22.48	<0.0001
*EZ_TV_* (%)^†^	1.614 ± 0.59	0.969 ± 0.54	<0.0001	1.586 ± 0.59	0.955 ± 0.51	<0.0001
*EZ_Th_* (µ*m*)^†^	17.504 ± 2.09	14.579 ± 7.84	<0.0001	17.700 ± 3.36	13.972 ± 6.99	<0.0001
*OPL_EZ* (µ*m*)^†^	133.236 ± 18.29	73.811 ± 23.28	<0.0001	130.992 ± 23.82	74.192 ± 20.83	<0.0001
*ELM_EZ* (µ*m*)^*^	37.723 ± 5.98	6.404 ± 10.38	<0.0001	38.461 ± 6.14	8.189 ± 11.17	<0.0001
*BM_EZ* (µ*m*)^†^	56.591 ± 7.08	34.870 ± 7.25	<0.0001	58.019 ± 7.29	34.912 ± 5.09	<0.0001
*ELM_BM* (µ*m*)^†^	94.311 ± 12.49	18.947 ± 28.19	<0.0001	96.597 ± 12.16	22.806 ± 29.75	<0.0001
Pediatric patient group versus adult patient group						
*rEZI* (%)^*^	42.389 ± 23.26	36.108 ± 20.66	<0.1007	39.997 ± 22.58	36.177 ± 22.51	<0.3110
*EZ_TV_* (%)^†^	1.395 ± 0.58	<0.969 ± 0.53	<0.0001	1.293 ± 0.46	<0.965 ± 0.51	<0.0001
*EZ_Th_* (µ*m*)^†^	16.267 ± 4.60	14.560 ± 7.92	<0.0624	16.149 ± 4.63	13.935 ± 6.95	<0.0117
*OPL_EZ* (µ*m*)^†^	91.920 ± 17.70	73.215 ± 23.17	<0.0001	90.757 ± 17.30	73.833 ± 20.73	<0.0001
*ELM_EZ* (µ*m*)^*^	23.020 ± 6.90	6.182 ± 10.26	<0.0001	22.925 ± 7.18	8.132 ± 11.12	<0.0001
*BM_EZ* (µ*m*)^†^	38.518 ± 6.95	34.788 ± 7.15	<0.0019	38.656 ± 6.91	34.856 ± 5.02	<0.001
*ELM_BM* (µ*m*)^†^	61.029 ± 13.51	18.289 ± 27.91	<0.0001	59.661 ± 15.43	22.633 ± 29.61	<0.0001

* Wilcoxon rank-sum test.

† *t* Test,

The scans were analyzed at 0.5 mm nasal and temporal to the foveal center. Data are presented as mean ± SD. *P* values were obtained using the *t* test when data met the Shapiro–Wilk test for normality; otherwise, the Wilcoxon test was applied.

Each comparison evaluates biomarkers such as *rEZI*, *EZ_TV_*, *EZ_Th_*, and weighted peak distances, providing the mean, SD, and *P* values for nasal and temporal regions at 0*.*5 mm from the foveal center. Notable differences are observed between groups for various biomarkers. For instance, significant discrepancies in rEZI are noted between the control and pediatric groups (nasal, *P* < 0.0013; temporal, *P* < 0.0008), similar to findings between the control and adult groups.

However, when comparing pediatric and adult groups, no significant difference in rEZI is found (nasal, *P* < 0.1007; temporal, *P* < 0.3110). These results suggest that, although significant differences in rEZI are observed between healthy controls and both pediatric and adult *RPE65*-IRD patients, there is no significant difference in rEZI between pediatric and adult patients. This finding implies that rEZI values may stabilize or change differently with age within the *RPE65*-IRD cohort.

The Pearson correlation analysis reveals several key relationships. In the first set, *rEZI* shows a moderate positive correlation with *EZ_TV_* (*r* = 0.414). It is also moderately correlated with ELM_BM (*r* = 0.329), while displaying a weak negative correlation with *EZ_Th_* (r = −0.085). In the second set, *EZ_TV_* demonstrates moderate positive correlations with *OPL_EZ_* (r = 0.333), and a weak positive correlation with *EZ_Th_* (r = 0.107). Notably, ELM_EZ shows almost no correlation with *EZ_TV_* (r = −0.006). These results highlight the complex relationships among biomarkers, *rEZI*, and *EZ_TV_* in *RPE65*-IRD. For a complete analysis of all correlations, we refer the reader to the Supplementary Material.

### Longitudinal Analysis After Treatment With VN

As the patients are followed for at least 5 years after VN therapy according to the requirements of the PERCEIVE registry imposed by the European Medicines Agency EMA (EUPAS31153, https://catalogues.ema.europa.eu/node/3021/data-management). More data can be collected at follow-up. As a general observation, the biomarkers showed fewer deviations in younger patients than in older patients compared with healthy controls. Exceptions were related to differences in phenotype severity, indicating some genotype–phenotype correlation of *RPE65*-IRDs. Unilaterally treated patients tended to have lower values both in their treated and untreated eyes. This worse performance was usually the reason for treating only one eye.

**Figure 4.**
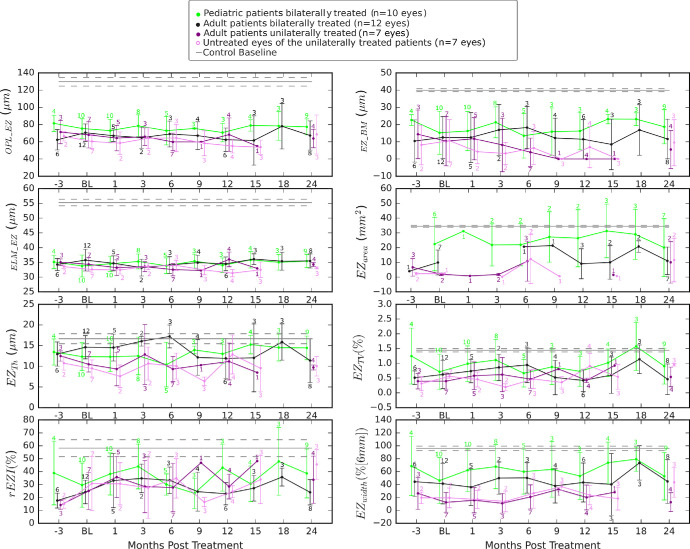
A comprehensive longitudinal analysis spanning −3 to 24 months after treatment of *RPE65*-IRD. The data are based on the results reported in.^19^ The numbers refer to the number of eyes per visit.

Longitudinal analysis (Fig. 4^5^) of biomarkers after treatment with VN revealed significant trends in pediatric and adult patients. Pediatric patients P05 and P04 showed consistent increases in outer retinal layer thicknesses (*OPL_EZ*, *ELM_EZ*) and EZ area over 24 months. P05 improved steadily in both *OPL EZ* and *ELM_EZ*, indicating a positive treatment response. Conversely, adult patients such as P16 and P02 exhibited varying responses, with differences in EZ thickness (*BM_EZ*) and variability (*EZ_TV_*) between eyes. P16 showed moderate improvements in *EZ_Th_*, whereas P02 consistently improved in both *EZ_Th_* and *EZ_TV_*. Untreated patients (UP01, UP03) demonstrated stable biomarker levels. Individual patient results are provided in the Supplementary Material.

**Figure 5.**
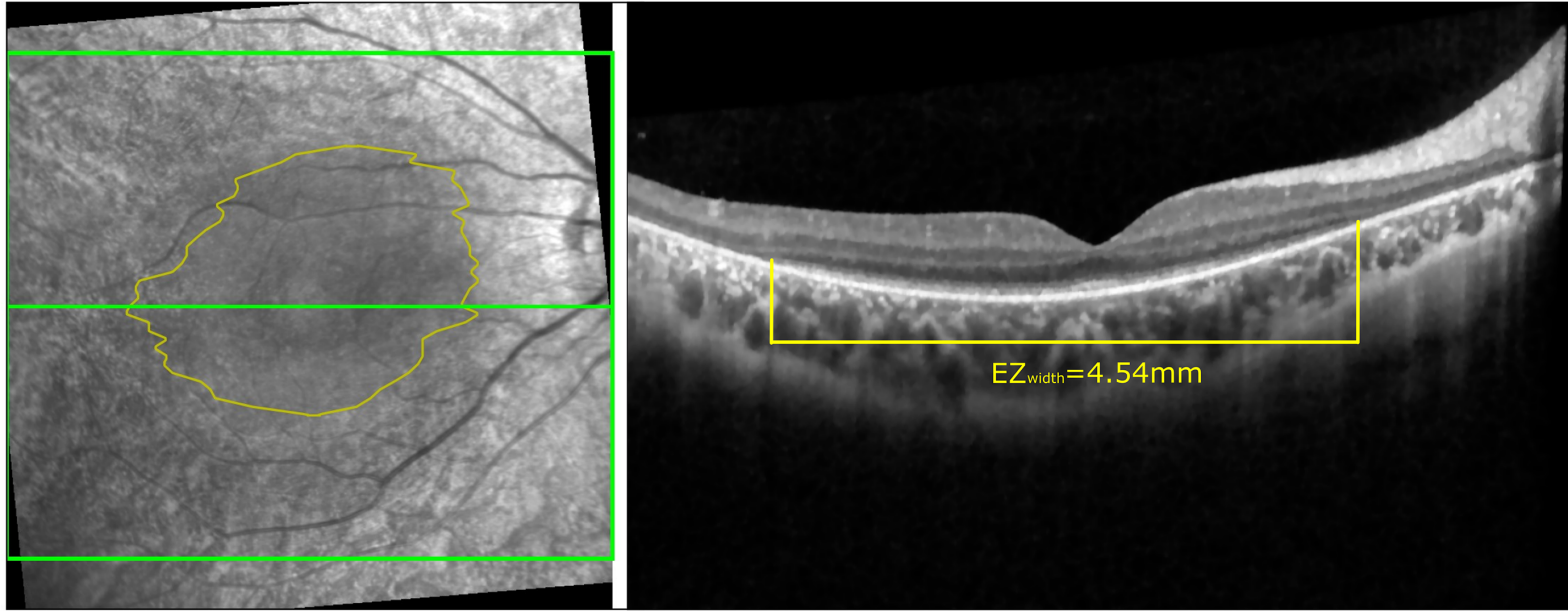
Automated segmentation was used to obtain the EZ area in volume scans focused on region of interest Early Treatment Diabetic Retinopathy Study at 6 mm. For instance, in the right eye of Patient P04, analysis involved 60 B-scans with 1536 A-scans each. At baseline, the patient exhibited a best-corrected visual acuity of 0.5 logMAR, with an EZ area measuring 15*.*15 mm2 (refer to Lorenz et al.^19^). Refer to Table S2 for comprehensive numerical results regarding EZ area and EZ diameter for each *RPE65*-IRD patient.

### EZ Area and EZ Width

In our quantitative analysis, we emphasize the significance of the EZ area as a crucial biomarker derived from SD-OCT scans.^11^ The process involves precisely measuring its width for each B-scan. Our study, comprising 96 SD-OCT scans (22 from healthy controls and 74 from patients with *RPE65*-IRD), underscores the pivotal role of EZ area size within the region of interest. These measurements serve as quantitative indicators of retinal structural changes and can be correlated with visual field measurements (data not shown). It is important to note that the EZ area limits are calculated exclusively where the EZ exists. Subsequently, we use linear interpolation to fill gaps between B-Scans. Finally, pixels within the EZ limits are tallied and scaled to square millimeters using the digital pixel size (approximately 121 µm^2^ according to the Spectralis manual). Figure 5 provides an example of the EZ area and its width on the foveal center B-scan.

As a result, significant differences were observed: the control group exhibited an EZ area of 34*.*476 ± 0*.*66 mm^2^, whereas the pediatric *RPE65*-IRD group showed 27*.*119 ± 14*.*30 mm^2^ and the adult *RPE65*-IRD group had an EZ area of 8*.*110 ± 8*.*88 mm^2^. The *P* value for the comparison between the control group and the pediatric *RPE65*-IRD group was 0*.*0170, whereas, for both the adult and pediatric *RPE65*-IRD groups against the control group, the *P* value was less than 0*.*0001.

**Figure 6.**
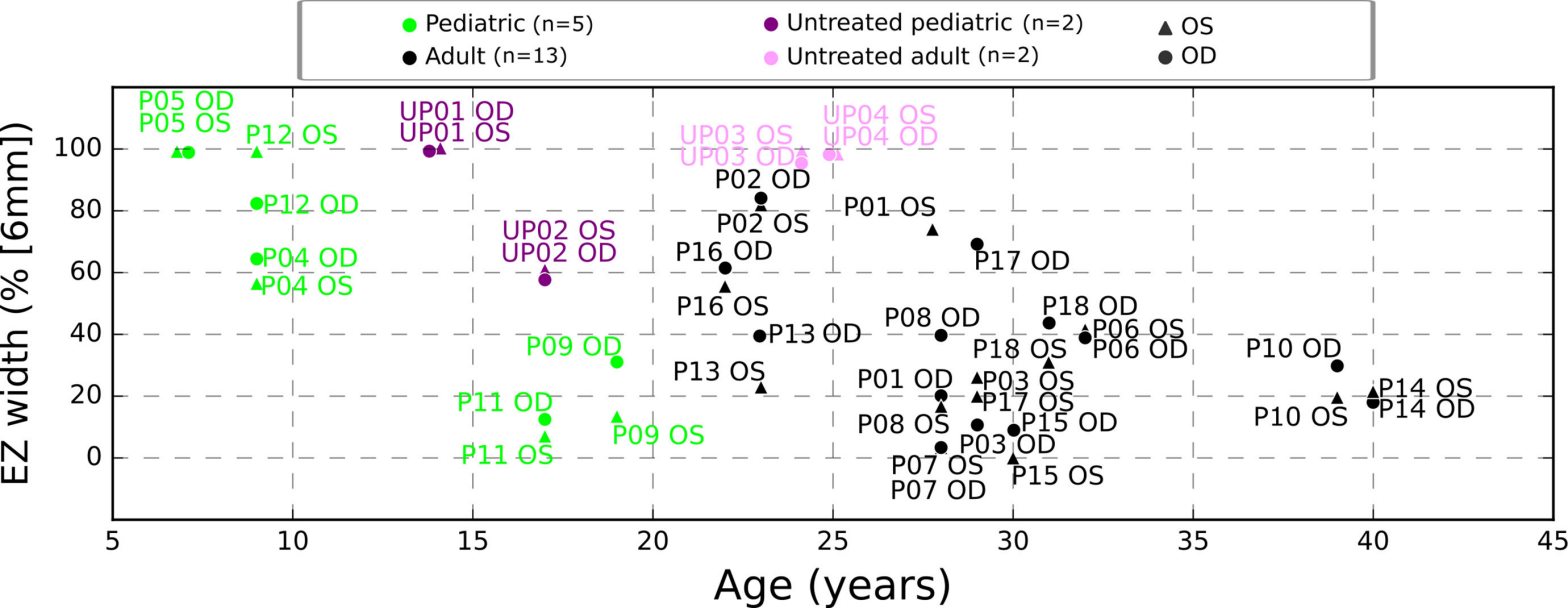
Relationship between age and EZ width expressed as a percentage (%) of the region of interest. Pediatric patients (*<*20 years) and adult patients are divided into treated (P) and untreated (UP) groups, with data marked for each eye (left and right). All measures of the treated eyes are taken at baseline. A trend can be observed of larger EZ width at younger age. Older patients with still relatively large EZ width reflect phenotypic differences, with some patients presenting milder disease. This knowledge is important for guiding treatment decisions.

Figure 6 illustrates age-EZ width correlation, grouped by pediatric (*<*20 years) and adult categories, and further subdivided into treated (P) and untreated (UP) groups. Each data point represents left and right eyes, facilitating comparisons across age and treatment. Analysis indicates that untreated patients mostly exhibit EZ widths between 80% and 98% of the region of interest, suggesting larger widths reflecting less severe or less progressed phenotypes, which was a factor for deferring treatment in such patients. Moreover, most adult patients show lower EZ widths compared with pediatric ones, except for patients P09 and P11, whose widths closely align with 0% to 20%. Of note, P09 and P11 had severe nystagmus hampering reproducible SD-OCT scans, and impeding both morphological and functional characterization of the disease stage.

## Discussion

In this work, we have proposed for the first time several novel concepts to quantitatively assess differences between *RPE65*-IRD patients and healthy controls in the outer retina imaged with SD-OCT with a focus on the EZ. Most of the biomarkers we introduced exhibited statistically significant differences. Despite the limited number of scans, the methods we used resulted in quite robust results showing the validity of our approach of DL to allow time-efficient quantitative biomarker analysis. Interestingly, the most significant differences were observed in the four weighted peak distances. In the future, these novel biomarkers could be correlated with visual function including best-corrected visual acuity and mesopic retinal sensitivity tested with microperimetry, as well as multifocal ERG measurements. The biomarkers quantifying the thickness of the EZ or its total variation were inferior indicators of *RPE65*-IRD in the pediatric group, but became significant in adults. Most of the novel biomarkers were changed significantly already early on, indicating that significant changes in the morphology of the outer retina may be present at birth or shortly afterward, in particular of the inner and outer segments and the outer nuclear layer. This questions up to which degree any therapy can reverse or prevent these changes, even if applied very early. An abnormal structure of EZ without further quantification has been observed in young children with SD-OCT. The data are in line with histology data of the retina of Briard dogs with a naturally occurring 4-bp deletion in *RPE65*.^27^ In any case, these novel biomarkers can be an additional way to monitor the in vivo treatment effects in patients and eventually also in animal studies as a noninvasive method. The longitudinal data we show have the problem of small numbers at the various time points after treatment impeding meaningful statistical analysis. This reflects the incomplete data collection of retrospective studies without a strict protocol such as the PERCEIVE register (EUPAS31153, https://catalogues.ema.europa.eu/node/3021/data-management), and variable compliance and logistics problems of the patients and their families to come to the recommended follow-up Examinations. Some of the patients live far away from the treatment site at the Department of Ophthalmology in Bonn. We confirm that EZ diameter and EZ area are valuable objective biomarkers of centripetal disease progression typical of rod–cone IRDs. Our novel approach allows quantification of both biomarkers within an acceptable time frame, thus enabling correlation with visual field measurements and quantification of the natural history of the disease in a large cohort of patients, as well as the definition of optimal timing of available and future treatments and treatment monitoring.

A strength of our study is the precise segmentation of the outer retinal layers guaranteed by the DL-based algorithm with its relatively high dice similarity coefficient and sensitivity throughout the entire cohort. This is a prerequisite for the exact quantification of the nine biomarkers we identified as meaningful parameters to describe with high resolution the outer retina in *RPE65*-IRD.

A limitation of our study is the relatively small data set of patients with *RPE65*-IRD and the available SD-OCT scans. In addition, 9 of the 44 eyes have high myopia (at least −5 diopter SER). However, according to the literature, the effect of high myopia on the outer retina is less significant than on the inner retina, and published data show a wide range of layer thicknesses for a wide range of refraction and ocular lengths.^28^ These uncertainties, however, still allow monitoring of individual disease courses with and without therapy. To further validate our algorithm, including more patients with *RPE65*-IRD is desirable. Further, to analyze how specific the novel biomarkers on EZ internal structure are, testing patients with other forms of IRDs will be interesting.

## Conclusions

Our study presents an automated DL-based approach for the precise evaluation of retinal changes in RPE65-associated inherited retinal dystrophy (*RPE65*-IRD) including treatment-related effects using OCT (SD-OCT) data. This method effectively overcomes the laborious and specialized nature of manual SD-OCT layer segmentation. We conducted a thorough quantitative analysis of nine biomarkers within the EZ, revealing significant differences between *RPE65*-IRD patients and healthy controls, notably in intensity profiles of EZ with the other layers of the outer retina and the volumetric area of EZ. Owing to the limitations of our study, a major use of our algorithms is the monitoring of individual disease courses.

## Supplementary Material

Supplement 1
