# Supplementary material for: Deep Learning–Based SD-OCT Layer Segmentation Quantifies Outer Retina Changes in Patients With Biallelic RPE65 Mutations Undergoing Gene Therapy
Source: Invest Ophthalmol Vis Sci. 2025 Jan 2;66(1):5. doi: 10.1167/iovs.66.1.5 (PMC11702825; doi:10.1167/iovs.66.1.5)
Supplement: Supplement 1 [file iovs-66-1-5_s001.pdf]

Supplementary Material: Deep learning-based SD-OCT layer segmentation quantifies outer retina changes in patients with biallelic RPE65 mutations undergoing gene therapy

German Pinedo-Diaz[†1], Birgit Lorenz[†2, 3], Sandrine H. Künzel[2], Sarah Thiele[2, 4], Susana Ortega-Cisneros[1], Eduardo Bayro Corrochano[1], Frank G. Holz[2], and Alexander Effland[5]

[1]Center for Research and Advanced Studies, Cinvestav, Zapopan, Mexico

[2]Dept of Ophthalmology, University Hospital, Bonn, Germany

[3]Transmit Centre for Translational Ophthalmology, c/o Justus-Liebig-University Giessen, Giessen, Germany

[4]Dept of Ophthalmology, University Medical Center Hamburg-Eppendorf, Hamburg, Germany

[5]Institute for Applied Mathematics, University of Bonn, Bonn, Germany

[†]Contributed equally

A Supplementary Material

A.1 Literature Review

There has been a substantial amount of methods for the automatic segmentation of retinal layers in SD-OCT and SD-OCT images. Some of the earliest methods for segmenting retinal layers in SD-OCT images were proposed by Garvin et al.[1] and Ehnes et al.[2] These methods used a graph theory and machine learning approach to extract features from the SD-OCT images, which were then used to train a classifier to segment the retinal layers. Bekalo et al.[3] proposed a multi-stage approach for accurately segmenting retinal layers in SD-OCT images 3-D volume scans. This method includes pre-processing steps, the graph-cut-based initial estimation of layer boundaries, and refinement using a CNN. Yazdanpanah et al.[4] used an active contour-based method for segmenting intra-retinal layers in SD-OCT images, which combines an intensity-based active contour model with a shape-based regularization term.

In recent years, DL-based approaches have been presented with promising outcomes. For instance, Venhuizen et al.,[5] Liu Xiaoming et al.,[6] and Maloca et al.[7] proposed a method for segmenting macular layers in SD-OCT images using a DL framework, and Mishra et al.[8] with a graph-based and DL method. Additionally, Roy et al.[9] proposed RelayNet which is a fully convolutional network to achieve pixel-wise segmentation of retinal layers. Likewise, Liu et al.[10] proposed a method for segmenting retinal layers in SD-OCT images using multi-scale DL. The method uses a multi-scale CNN to extract features from the SD-OCT images. Then the features are used to train a classifier to segment the retinal layers. Cazanas-Gordon et al.[11] introduce a novel deep-learning architecture for accurate boundary segmentation of retinal layers and macular cystoid edema. The proposed Multi-scale Attention Gated Network (MAGNet) combines the benefits of attention mechanisms and gated units to enhance feature representation and capture contextual information at multiple scales. Qiaoliang Li et al.[12] utilize the Xception65 network to extract and learn the characteristics of retinal layers, which incorporates a spatial pyramid pooling module to capture multi-scale feature information. Fazekas et al.[13] SD-LayerNet leverages a semi-supervised learning framework, combining labeled and unlabeled data. The network incorporates a disentangled representation learning module, which focuses on separating anatomical and imaging-specific features, aiding in better understanding the underlying structure of retinal layers. By contrast, a novel method presented by Farshad et al.,[14] introduces the Y-Net architecture consisting of two encoders, one for spatial information and the other for spectral details, which capture both structural and contextual features of the input image. These encoders are combined through a fusion block to effectively integrate spatial and spectral information. The Y-Net

is trained using a combination of supervised and unsupervised learning, where the supervised loss ensures accurate segmentation and the unsupervised loss encourages consistency in the learned representations.

A.2 Dataset and Data Processing

Time categories were set to cover data at baseline (BL, 0 d), month 1 (M1, <42 d.p.t. (days past treatment)), month 3 (M3, 42 to 131 d.p.t.), month 6 (M6, 132 - 221 d.p.t.), month 9 (M9, 222 - 310 d.p.t.), month 12 (M12, 311 - 406 d.p.t.), month 18 (M18, 407 - 582 d.p.t), month 24 (M24, 582 - 771 d.p.t.). In case more than one visit per eye was available in a category, the latest visit of a category was included (see also Lorenz et al.[15]).

A.2.1 Data Augmentation and Normalization

Given the limited availability of training data, we employed data augmentation, a well-known technique that helps to prevent overfitting and enhances the generalization capability of our DL model. Data augmentation involves applying various transformations to the existing data and creating additional samples with realistic variations. We grouped the augmentation methods into two categories: intensity transforms and geometric adjustments, and applied them randomly during training. Intensity transforms were exclusively applied to the SD-OCT B-Scans. These transformations focus on altering the pixel intensity values while preserving the underlying structures. By contrast, geometric adjustments were applied to both the segmentation masks and their corresponding B-Scans.

- Random intensity transformations applied only to B-Scans:
  - random color-jitter modifying brightness, contrast, and saturation (for each B-Scan, random values within the range of [-0.5, 0.5] are independently drawn, the probability of applying this transformation is 50%);
  - random contrast-limited adaptive histogram equalization with clip limit of 2 and tile grid size of 4×4;
  - random non-linear gamma adjustment ($\mathcal{P}_{i,j} = \mathcal{P}_{i,j}^{\gamma}$) with $\mathcal{P}_{i,j}$ denoting the pixel value indexed in x and y-direction (γ values are chosen from a uniform distribution in the range $(0.7, 1.3)$);
  - random Gaussian blur with a probability of 20% and standard deviation 0.1 clipped to the interval $(0, 1)$.
- Random geometric transformation SD-OCT B-Scans and masks:

- random rotations to the images and masks uniformly drawn from the interval $(-25°, 25°)$;
- random horizontal flips to the images and their associated masks with a probability of 50%;
- elastic deformation with a random square deformation. In detail, with a probability of 30%, a random square deformation using 8 nodes along the x- and y-axis is applied. The shift region is uniformly sampled from the interval $(0.8, 1.5)$ from a normal distribution with a zero mean and standard deviation at each node.

For better inter- and intra-patient analysis, the following data normalization was performed:

$$I_{\text{norm}}(i,j) = \frac{I(i,j)}{q_{95\%}},$$

where $I_{\text{norm}}(i,j)$ represents the normalized pixel value at indices i and j of the B-Scan matrix I. The value $q_{95\%}$ is the 95th percentile value.

A.2.2 Training Setup

The training was performed on a dedicated high-performance system with Ubuntu 20.04 operating system, 64 RAM memory, 4x48GB VRAM NVIDIA A40 DataCenter GPU, and PyTorch framework.

The Adam optimizer is used with a hyper-parameters setup with a learning rate of 0.001, weight attenuation of 10^{-4} step learning scheduler of 80% of learning rate every 50 epochs. Furthermore, the momentum variables are $\beta_1 = 0.8$ and $\beta_2 = 0.999$. We use a batch size of 128 and 300 epochs. Besides, the loss function used for all experiments is the Dice-Cross-Entropy (see equation 1) with $\lambda = 0.6$.

The dice similarity coefficient (DSC) and Sensitivity (Se) are metrics used to assess the performance of our model:

$$\text{DSC} = \frac{2\text{TP}}{2\text{TP} + \text{FP} + \text{FN}}, \qquad \text{Se} = \frac{\text{TP}}{\text{TP} + \text{FN}},$$

where TP represents the true positives classification, FP are the false positives, and FN represents the false negatives. First, the DSC measures the agreement between predicted and ground truth segmentation masks, quantifying the accuracy of boundary delineation. A higher DSC indicates better segmentation performance. Sensitivity, on the other hand, evaluates the model's ability to detect true positive instances, indicating its capacity to capture desired features.

Table S1 summarizes training results from validation using the UKB dataset, divided into UKB Control, UKB *RPE65*-IRD, and UKB Combined datasets. For UKB Control, representing healthy subjects, the

average Dice Similarity Coefficient (DSC) was 0.9163, with individual retinal layer DSC values ranging from 0.8609 to 0.9953. Conversely, the UKB *RPE65*-IRD dataset, comprising individuals with RPE65-associated inherited retinal dystrophy, had an average DSC of 0.8781, with varying DSC values across layers. Combining both datasets in UKB Combined yielded an average DSC of 0.9238, showing improved segmentation. In addition, Sensitivity (Se) values followed similar trends.

Table S1: Training results of the validation using UKB dataset exploiting the U-Net-based model. The training datasets are UKB controls for the segmentation of healthy subjects (first two rows), *RPE65*-IRD patient data (third/fourth row), and a combination of both datasets mentioned above (last two rows). We refer the reader to the caption of Figure 1 for the abbreviations.

**Dataset**	**Metric**	**Retinal Layers**					**Average**
		**BG**	**EZ**	**OPL**	**ELM**	**IZ-RPE-BM**	
UKB Control (n=94)	DSC	0.9953	0.8920	0.8609	0.8995	0.9338	0.9163
	Se	0.9940	0.9081	0.9032	0.9105	0.9341	0.9300
UKB *RPE65*-IRD (n=23)	DSC	0.9943	0.8239	0.7878	0.8611	0.8889	0.8712
	Se	0.9936	0.7839	0.8249	0.8833	0.9043	0.8781
UKB Combined (n=117)	DSC	0.9954	0.9051	0.9028	0.8875	0.9283	0.9238
	Se	0.9944	0.9122	0.9655	0.8218	0.9497	0.9287

A.2.3 Total Variation Denoising

Total Variation (TV) denoising reduces image noise while preserving the original image.[16] We use Chambolle's iterative algorithm[17] to denoise SD-OCT images with different regularization parameters α and assess EZ granularity with local variation. The TV denoising problem can be formulated as an optimization problem, where the goal is to find a denoised image that minimizes the total variation of the function while being close to the observed noisy data. For 2-D images, the discretization of TV reads as

$$\mathrm{TV}(u) = \sum_{i,j} \sqrt{(\nabla_x u_{i,j})^2 + (\nabla_y u_{i,j})^2}, \tag{1}$$

where ∇_x and ∇_y are forward derivatives in horizontal and vertical directions, respectively.

When provided with input data, typically a noisy image denoted as f, the objective of total variation denoising is to discover an image u that possesses a lower total variation than f, while still maintaining a degree of similarity to f. This concept is formally encapsulated within the Rudin–Osher–Fatemi (ROF) minimization problem:

$$\min_u \sum_{i,j} \left(\mathrm{TV}(u) + \frac{1}{2\alpha}(f_{i,j} + u_{i,j})^2 \right), \tag{2}$$

where α is the denoising weight.

A.3 Statistical Analysis

The analysis of biomarker data, including thickness, granularity, reflectivity, and relative intensity of the ellipsoid zone (EZ), was conducted using both QQ plots and the Shapiro-Wilk test to assess normality. The QQ plot indicates that most of the biomarker data closely follows the reference line, suggesting approximate normality for many variables (see Figure S1).

However, the Shapiro-Wilk test results provide a more detailed assessment of normality. For the biomarkers $rEZI$ and EZ_ELM, the p-values are 0.0067 and less than 0.0001, respectively, indicating deviations from normality. On the other hand, biomarkers EZ_{TV} ($p < 0.2389$), EZ_{Th} ($p < 0.6285$), EZ_OPL ($p < 0.5376$), and EZ_BM ($p < 0.7403$) show p-values greater than 0.05, suggesting that these variables are normally distributed.

Considering the dataset of 22 patients and 33 eyes, with some cases where ELM and/or EZ are not detected, these results guide the choice of statistical tests. For biomarkers that are approximately normally distributed, parametric tests like the t-test are appropriate. For $rEZI$ and EZ_ELM, which do not follow a normal distribution, non-parametric tests Wilcoxon test was used.

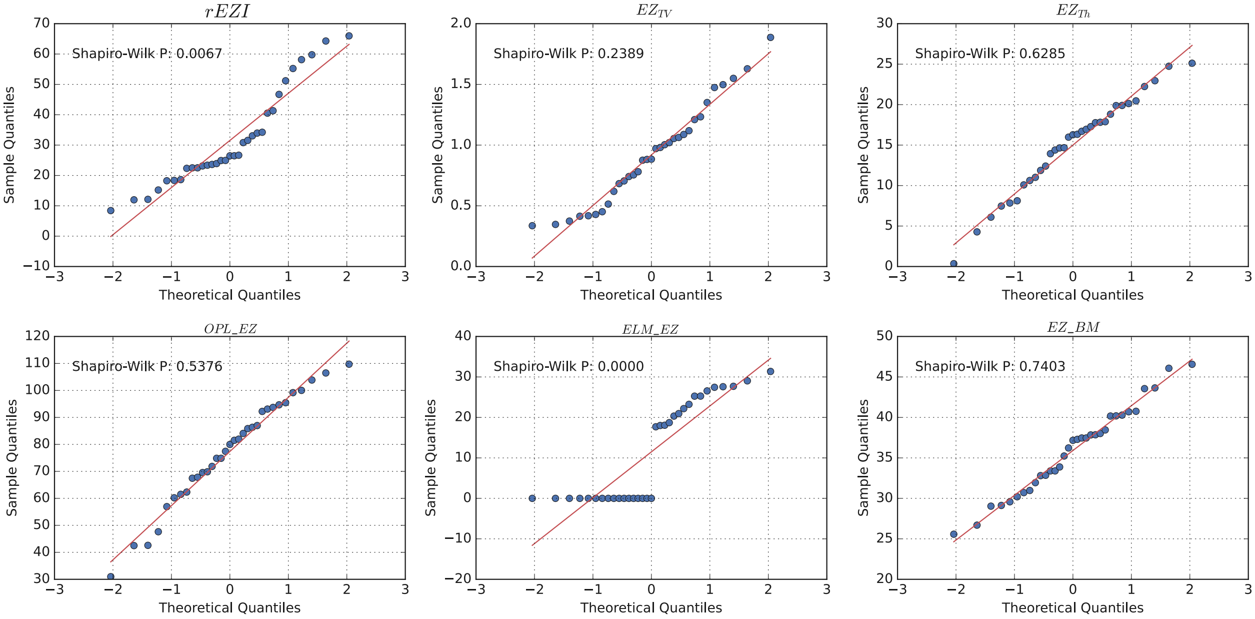

Figure S1: QQ plots and Shapiro-Wilk test results for biomarker data. The QQ plots suggest normality for most biomarkers.

The supplementary Figure S2 displays the correlation matrix, providing a comprehensive view of the relationships between various biomarkers.

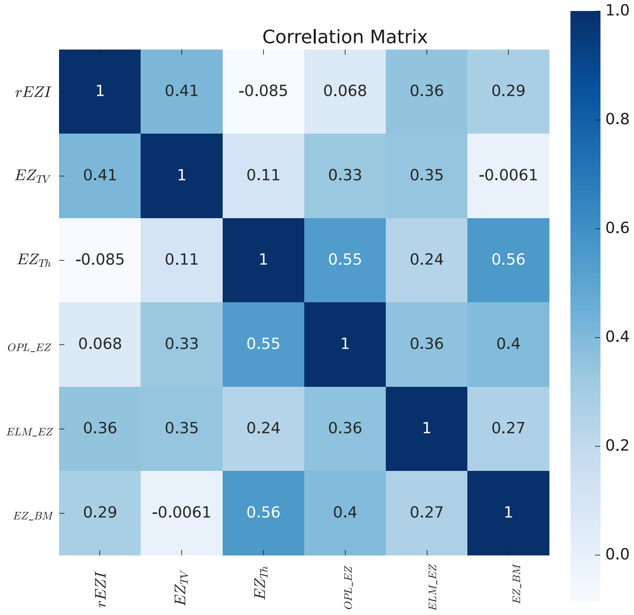

Figure S2: Correlation matrix of EZ biomarkers. Each cell displays the Pearson correlation coefficient, showing the strength and direction of relationships between biomarkers. Positive values indicate direct correlations and negative values show inverse correlations.

A.4 Longitudinal Analysis

Table S2 and Figure 4 show the longitudinal course following VN therapy of all biomarkers defined in this study. No major changes were seen for all parameters applied, although in some exceptional eyes, the values were different at follow-up, resulting in both worsening and improvement.

A.5 Refractive Status

The refractive status in Table S3 details the refractive errors for each patient in both the right eye (OD) and the left eye (OS).

Table S2: Biomarkers per patient from baseline up to 24 months after treatment with VN (Mean±Standard deviation). Divided by pediatric and adult, and patient PXX for patients with treatment, UPXX for untreated patients. All measures were taken $0.5mm$ nasal or temporal of the fovea as more peripheral locations had already lost some of the outer retinal layers. a) Shows all quantities of (2), and EZ_{area}. b) Shows EZ_{Th} (3), EZ_{TV}(6), $rEZI$ (4), and EZ_{width}. Coded patient numbers are the same as in Lorenz et al.[15] (M male, F female). Age is given in years. EZ area values are only computed for eyes if volume scans are available.

(a)

Patient	Sex (M: Male F: Female)	Age (years)	Eye	T[1]	*OPL_EZ* (μm)		*ELM_EZ* (μm)		*EZ_BM* (μm)		EZ_{area} (mm^2)	
					**BL[2]**	**M_T[3]**	**BL**	**M_T**	**BL**	**M_T**	**BL**	**M_T**
**Pediatric**												
P05	M	7	OS	15	96.0 ± 4.1	96.7 ± 5.43	25.22 ± 1.09	27.25 ± 0.63	38.54 ± 0.93	37.46 ± 1.74	41.96	43.58
			OD	18	103.63 ± 5.32	109.45 ± 8.31	25.79 ± 0.63	26.5 ± 0.35	40.11 ± 2.27	36.82 ± 0.67	41.58	41.36
P04	M	9	OS	12	85.4 ± 6.8	68.63 ± 1.49	25.22 ± 1.7	24.37 ± 1.33	35.24 ± 2.83	33.43 ± 0.05	8.87	12.7
			OD	9	88.16 ± 6.74	87.49 ± 6.52	26.5 ± 1.39	26.37 ± 1.72	34.64 ± 1.55	33.85 ± 1.37	9.61	15.15
P12	M	9	OS	12	82.65 ± 0.98	75.13 ± 1.54	20.88 ± 1.52	16.84 ± 6.25	36.21 ± 0.47	34.93 ± 0.67	35.69	40.07
			OD	18	78.87 ± 2.76	60.78 ± 4.24	18.71 ± 2.07	18.73 ± 1.69	35.85 ± 1.44	31.87 ± 1.41	31.4	35.94
P11	F	17	OS	15	72.53 ± 4.08	79.06 ± 2.14	14.03 ± 7.08	17.93 ± 1.39	34.06 ± 0.46	34.75 ± 0.59		
			OD	9	84.98 ± 3.48	67.85 ± 4.68	0.0	0.0	40.1 ± 0.78	35.08 ± 1.01		
P09	M	19	OS	24	80.67 ± 2.72	69.97 ± 4.14	30.42 ± 0.47	3.87 ± 5.48	34.37 ± 0.49	36.86 ± 1.36	1.02	1.11
			OD	12	76.81 ± 3.84	83.66 ± 1.21	25.2 ± 0.08	20.25 ± 0.98	35.55 ± 0.44	36.22 ± 0.82		
**Adult**												
P16	M	22	OS	24	72.18 ± 1.41	65.03 ± 4.97	18.29 ± 0.61	9.98 ± 6.02	33.09 ± 0.85	33.2 ± 0.4	16.62	19.95
			OD	UT								
P02	F	23	OS	9	99.14 ± 6.3	106.09 ± 6.03	27.24 ± 1.65	28.15 ± 2.3	33.8 ± 0.74	37.77 ± 1.14	18.61	21.38
			OD	12	93.13 ± 7.8	100.74 ± 7.7	25.89 ± 1.49	28.34 ± 1.55	36.12 ± 2.16	39.88 ± 1.25	15.15	17.05
P13	F	23	OS	12	47.76 ± 3.59	84.74 ± 7.53	0.0	0.0	29.96 ± 0.76	37.68 ± 3.14		
			OD	UT								
P01	M	28	OS	12	66.4 ± 3.44	63.91 ± 6.84	19.74 ± 2.14	18.18 ± 2.7	33.94 ± 1.71	34.47 ± 1.73	18.4	20.31
			OD	12	54.4 ± 14.82	48.11 ± 3.58	27.32 ± 0.25	19.97 ± 0.3	33.2 ± 1.75	33.88 ± 1.32	5.21	6.54
P07	F	28	OS	12	73.99 ± 0.43	46.97 ± 2.73	0.0	0.0	43.34 ± 0.11	33.96 ± 0.82	0.66	0.46
			OD	15	66.91 ± 3.5	39.13 ± 1.39	0.0	0.0	43.17 ± 1.75	33.64 ± 0.36	1.38	1.94
P08	M	28	OS	12	82.56 ± 7.18	70.04 ± 5.42	18.97 ± 0.13	0.0	35.2 ± 1.37	35.92 ± 0.78		
			OD	9	82.53 ± 7.22	65.25 ± 2.56	20.63 ± 0.16	0.0	36.36 ± 1.1	31.87 ± 0.53		
P03	M	29	OS	24	73.27 ± 6.22	53.35 ± 3.17	22.23 ± 1.77	0.0	35.74 ± 0.8	34.69 ± 0.42	1.51	1.01
			OD	UT								
P17	F	29	OS	12	78.29 ± 13.29	61.77 ± 3.67	0.0	11.13 ± 1.87	33.19 ± 1.71	32.07 ± 0.43		
			OD	9	58.58 ± 16.14	55.13 ± 3.53	0.0	0.0	33.61 ± 2.78	32.39 ± 0.65		
P15	M	30	OS	12	88.16 ± 3.36	90.83 ± 3.77	21.72 ± 7.73	0.0	36.08 ± 0.94	34.3 ± 0.21		
			OD	UT								
P18	M	31	OS	UT								
			OD	12	60.19 ± 3.06	46.31 ± 1.24	0.0	0.0	34.03 ± 0.6	36.57 ± 1.3		
P06	M	32	OS	18	63.09 ± 5.9	55.98 ± 4.68	0.0	0.0	29.69 ± 1.21	31.75 ± 0.13		
			OD	12	58.98 ± 4.83	60.35 ± 1.92	0.0	0.0	32.21 ± 1.26	32.6 ± 0.41		
P10	F	39	OS	UT								
			OD	15	71.12 ± 8.51	77.39 ± 15.11	0.0	0.0	40.81 ± 3.56	33.85 ± 0.68	2.88	1.56
P14	F	40	OS	UT								
			OD	12	59.78 ± 5.4	67.11 ± 5.84	0.0	0.0	30.47 ± 0.57	30.39 ± 1.08		
**Untreated Patients**												
UP01	M	14	OS	UT	102.43 ± 8.13		22.14 ± 3.4		44.34 ± 1.72			
			OD	UT	99.12 ± 5.63		20.98 ± 1.58		43.62 ± 1.57			
UP02	F	17	OS	UT	95.61 ± 7.79		21.62 ± 1.71		36.81 ± 4.19			
			OD	UT	93.21 ± 6.5		19.68 ± 1.64		36.63 ± 3.72			
UP03	M	23	OS	UT	96.52 ± 4.25		25.86 ± 1.27		31.6 ± 1.34			
			OD	UT								
UP04	M	25	OS	UT	93.95 ± 2.29		28.72 ± 1.26		48.41 ± 2.89			
			OD	UT	91.7 ± 2.64		29.01 ± 1.47		47.95 ± 2.84			

[1]Date of the last visit

[2]Baseline treatment date

[3]Months post-treatment using the date of the last visit (9, 12, 15, 18, 24, or UT for untreated fellow eyes)

(b)

Patient	Sex (M: Male F: Female)	Age (years)	Eye	T[1]	EZ_{Th} (μm)		$EZ_{TV}(\%)$		$rEZI(\%)$		$EZ_{width}(\%[6mm])$	
					**BL**[2]	M_T[3]	**BL**	M_T	**BL**	M_T	**BL**	M_T
**Pediatric**												
P05	M	7	OS	15	16.78 ± 0.52	17.5 ± 0.42	1.77 ± 0.09	1.39 ± 0.01	79.87 ± 5.48	51.98 ± 2.73	99.25	99.25
			OD	18	18.97 ± 0.6	18.35 ± 0.44	1.29 ± 0.08	2.33 ± 0.1	55.37 ± 3.48	82.01 ± 5.33	98.9	98.9
P04	M	9	OS	12	14.96 ± 1.66	13.4 ± 0.97	0.99 ± 0.08	1.09 ± 0.24	22.07 ± 2.52	21.76 ± 0.4	56.49	70.6
			OD	9	15.48 ± 1.51	15.57 ± 1.54	1.26 ± 0.09	1.17 ± 0.13	27.29 ± 3.95	22.31 ± 0.91	64.42	77.03
P12	M	9	OS	12	10.94 ± 1.82	12.38 ± 1.29	1.49 ± 0.08	1.36 ± 0.1	47.77 ± 4.81	32.97 ± 1.75	99.23	95.9
			OD	18	11.07 ± 1.48	14.53 ± 2.05	0.96 ± 0.09	2.16 ± 0.28	28.19 ± 1.47	49.73 ± 2.91	82.36	77.63
P11	F	17	OS	15	10.7 ± 1.77	16.04 ± 1.05	0.31 ± 0.09	0.57 ± 0.11	14.81 ± 5.33	15.72 ± 0.29	7.07	28.91
			OD	9	15.65 ± 1.77	11.09 ± 2.78	0.3 ± 0.1	0.45 ± 0.1	17.61 ± 2.75	10.28 ± 1.39	12.48	15.33
P09	M	19	OS	24	10.71 ± 1.02	16.1 ± 4.96	0.74 ± 0.32	1.38 ± 0.63	32.13 ± 0.95	47.51 ± 13.69	13.49	19.50
			OD	12	11.69 ± 2.6	15.9 ± 0.86	0.49 ± 0.19	0.98 ± 0.38	27.46 ± 2.16	52.73 ± 1.22	31.06	29.05
**Adult**												
P16	M	22	OS	24	10.45 ± 1.93	7.18 ± 1.69	1.29 ± 0.3	0.63 ± 0.12	48.43 ± 0.62	36.49 ± 2.68	55.57	26.26
			OD	UT								
P02	F	23	OS	9	19.52 ± 1.95	22.08 ± 1.85	1.75 ± 0.16	1.94 ± 0.22	40.03 ± 1.84	48.8 ± 1.5	81.94	83.14
			OD	12	19.44 ± 3.67	20.45 ± 1.47	1.53 ± 0.07	1.18 ± 0.03	37.38 ± 2.79	34.6 ± 3.2	84.09	92.03
P13	F	23	OS	12	8.8 ± 0.77	16.24 ± 4.84	1.12 ± 0.31	0.8 ± 0.21	21.01 ± 0.85	43.37 ± 3.93	22.95	21.67
			OD	UT								
P01	M	28	OS	12	14.77 ± 0.53	11.65 ± 2.18	0.78 ± 0.07	0.77 ± 0.1	27.45 ± 10.35	46.01 ± 12.84	74.87	74.16
			OD	12	9.32 ± 3.29	4.94 ± 3.53	0.53 ± 0.06	0.16 ± 0.04	45.95 ± 25.95	8.67 ± 0.5	20.13	35.48
P07	F	28	OS	12	19.26 ± 0.44	12.13 ± 3.87	0.19 ± 0.12	0.18 ± 0.07	22.47 ± 2.0	43.37 ± 17.31	3.36	5.04
			OD	15	17.31 ± 1.47	3.52 ± 0.39	0.34 ± 0.16	0.36 ± 0.13	16.71 ± 2.29	25.08 ± 10.41	3.37	4.42
P08	M	28	OS	12	16.89 ± 1.94	5.45 ± 1.16	0.54 ± 0.13	0.41 ± 0.17	18.8 ± 1.78	15.99 ± 1.33	16.71	20.0
			OD	9	11.45 ± 2.04	8.74 ± 0.9	0.63 ± 0.25	0.33 ± 0.13	21.2 ± 1.64	11.64 ± 0.39	39.68	31.61
P03	M	29	OS	24	13.53 ± 3.46	14.3 ± 2.93	0.58 ± 0.17	0.55 ± 0.14	17.56 ± 1.27	24.3 ± 9.23	26.11	23.27
			OD	UT								
P17	F	29	OS	12	22.05 ± 2.47	18.05 ± 1.51	1.0 ± 0.06	0.63 ± 0.09	21.76 ± 2.18	19.74 ± 0.77	19.98	35.21
			OD	9	20.68 ± 3.39	9.71 ± 1.88	0.59 ± 0.21	0.42 ± 0.11	39.43 ± 13.41	27.58 ± 4.29	69.18	16.77
P15	M	30	OS	12	14.39 ± 1.85	10.2 ± 1.26	0.63 ± 0.13	0.74 ± 0.25	25.72 ± 1.36	30.29 ± 1.8	0.0	0.0
			OD	UT								
P18	M	31	OS	UT								
			OD	12	18.22 ± 2.75	9.6 ± 2.65	0.72 ± 0.07	0.39 ± 0.07	14.59 ± 2.3	16.41 ± 1.84	43.68	38.44
P06	M	32	OS	18	11.28 ± 0.64	13.67 ± 1.77	0.6 ± 0.14	0.94 ± 0.26	11.32 ± 1.06	42.67 ± 9.31	41.57	43.0
			OD	12	8.36 ± 2.62	11.94 ± 2.7	0.66 ± 0.17	0.41 ± 0.09	13.82 ± 0.72	19.55 ± 8.05	38.89	40.87
P10	F	39	OS	UT								
			OD	15	12.99 ± 3.74	13.74 ± 4.72	1.38 ± 0.33	1.39 ± 0.26	75.23 ± 10.99	69.89 ± 14.59	29.81	28.13
P14	F	40	OS	UT								
			OD	12	8.35 ± 1.58	10.54 ± 1.6	0.42 ± 0.06	0.47 ± 0.05	18.85 ± 7.44	65.52 ± 3.55	29.81	17.32
**Untreated Patients**												
UP01	M	14	OS	UT	20.01 ± 0.47		1.48 ± 0.09		27.19 ± 0.96		99.56	
			OD	UT	20.57 ± 1.01		1.25 ± 0.15		26.1 ± 0.49		99.56	
UP02	F	17	OS	UT	14.14 ± 2.58		0.87 ± 0.28		21.77 ± 3.12		60.91	
			OD	UT	12.07 ± 3.41		1.16 ± 0.46		34.76 ± 10.62		57.66	
UP03	M	23	OS	UT	14.39 ± 0.98		1.63 ± 0.14		30.63 ± 0.99		99.46	
			OD	UT								
UP04	M	25	OS	UT	19.37 ± 0.7		1.13 ± 0.19		54.78 ± 15.2		99.46	
			OD	UT	18.85 ± 0.58		1.29 ± 0.27		64.47 ± 12.55		99.45	

[1]Date of the last visit

[2]Baseline treatment date

[3]Months post-treatment using the date of the last visit (9, 12, 15, 18, 24, or UT for untreated fellow eyes)

Table S3: Ocular length and refractive status of both eyes for each patient. The refractive status includes spherical (SPH) and cylindrical (CYL) components, along with the axis (AXIS) of astigmatism.

Patient	Age	Ocular Length (R)	Ocular Length (L)	Refraction (R)	Refraction L	SER (R)	SER (L)
P005	7	21.441	21.41	+4,00 -2,25 170	+4,75 -2,75 4	2.875	3.375
P004	9	21.42	21.42	0,00 -1,75 7	+1,25 -1,50 174	-0.875	0.5
P012	9	-	-	+3,75 +2,25 5	+3,75 -2,75 167	4.875	2.375
P011	17	23.58	23.85	-2,0 -3,5 12	-2,75 -3,0 176	-3.75	-4.25
P009	19	23.08	22.91	-2 -4,5 6	-1,75 -3,75 175	-4.25	-3.625
P016	22	22.94	22.79	-2,75 -0,75 32	-1,5 -0,5 169	-3.125	-1.75
P002	23	23.2	22.68	-3,50 -1,50 171	-2,25 -1,00 14	-4.25	-2.75
P013	23	22.23	21.95	+2,25 -0,5 167	+3,75 -1 39	2	3.25
P001	28	24.47	25.83	-2,50 -1,75 83	-2,50 -1,75 107	-3.375	-3.375
P007	28	24.59	24.54	-6,25 -3,00 174	-5,50 -3,25 171	-7.75	-7.125
P008	28	22.4	22.51	+3,00 -1,25 24	+2,5 -2,25 167	2.375	1.375
P003	29	25.47	25.44	-8,25 -2,50 18	-8,00 -2,25 154	-9.5	-9.125
P017	29	22.15	22.18	+1 -1,25 156	+1,25 -1,25 27	0.375	0.625
P015	30	24.61	24.3	-3 -3 28	-4,25 -2,25 161	-4.5	-5.375
P018	31	22.71	23.1	+0,5 -3,25 12	+1,25 -4,75 171	-1.125	-1.125
P006	32	22.6	22.66	+4,00 -1,75 4	+4,25 -1,25 1	3.125	3.625
P010	39	23.92	23.9	-0,25 -2,50 167	-0,50 -2,25 2	-1.5	-1.625
P014	40	22.55	22.39	0 -1,5 25	+1 -6,75 152	-0.75	-2.375
UP01	14	-	-	+5,0 -2,0 0	+5,0 -2,0 0	4	4
UP02	17	-	-	-2,25 -2,0 163	-3,25 -2,0 13	-3.25	-4.25
UP03	23	-	-	-	-	-0.25	-0.5
UP04	25	25.82	25.86	-1,25 -2,25 160	-1,25 -2,25 170	-2.375	-2.375

References

[1] Garvin MK, Abramoff MD, Wu X, Russell SR, Burns TL, Sonka M. Automated 3-D Intraretinal Layer Segmentation of Macular Spectral-Domain Optical Coherence Tomography Images. *IEEE Trans Med Imaging.* 2009; 28(9): 1436-1447. doi: 10.1109/tmi.2009.2016958

[2] Ehnes A, Wenner Y, Friedburg C, et al. Optical Coherence Tomography (OCT) Device Independent Intraretinal Layer Segmentation. *Translational Vision Science & Technology* 2014; 3(1): 1. doi: 10.1167/tvst.3.1.1

[3] Bekalo L, Niu S, He X, et al. Automated 3-D Retinal Layer Segmentation From SD-OCT Images With Neurosensory Retinal Detachment. *IEEE Access* 2019; 7: 14894-14907. doi: 10.1109/access.2019.2893954

[4] Yazdanpanah A, Hamarneh G, Smith BR, Sarunic MV. Segmentation of Intra-Retinal Layers From Optical Coherence Tomography Images Using an Active Contour Approach. *IEEE Trans Med Imaging.* 2011; 30(2): 484-496. doi: 10.1109/tmi.2010.2087390

[5] Venhuizen FG, Ginneken vB, Liefers B, et al. Deep learning approach for the detection and quantification of intraretinal cystoid fluid in multivendor optical coherence tomography. *Biomed. Opt. Express* 2018; 9(4): 1545. doi: 10.1364/boe.9.001545

[6] Liu X, Fu T, Pan Z, et al. Automated Layer Segmentation of Retinal Optical Coherence Tomography Images Using a Deep Feature Enhanced Structured Random Forests Classifier. *IEEE J Biomed Health Inform* 2019; 23(4): 1404-1416. doi: 10.1109/jbhi.2018.2856276

[7] Maloca PM, Lee AY, Carvalho dER, et al. Validation of automated artificial intelligence segmentation of optical coherence tomography images. *PloS one.* 2019; 14(8): e0220063. doi: 10.1371/journal.pone.0220063

[8] Mishra Z, Ganegoda A, Selicha J, Wang Z, Sadda SR, Hu Z. Automated Retinal Layer Segmentation Using Graph-based Algorithm Incorporating Deep-learning-derived Information. *Sci Rep.* 2020; 10(1). doi: 10.1038/s41598-020-66355-5

[9] Roy AG, Conjeti S, Karri SPK, et al. ReLayNet: retinal layer and fluid segmentation of macular optical coherence tomography using fully convolutional networks. *Biomed. Opt. Express* 2017; 8(8): 3627. doi: 10.1364/boe.8.003627

[10] Liu TYA, Ling C, Hahn L, Jones CK, Boon CJF, Singh MS. Prediction of visual impairment in retinitis pigmentosa using deep learning and multimodal fundus images. *Br J Ophthalmol.* 2022; 107(10): 1484-1489. doi: 10.1136/bjo-2021-320897

[11] Cazanas-Gordon A, Silva Cruz dLA. Multiscale Attention Gated Network (MAGNet) for Retinal Layer and Macular Cystoid Edema Segmentation. *IEEE Access* 2022; 10: 85905-85917. doi: 10.1109/access.2022.3198657

[12] Li Q, Li S, He Z, et al. DeepRetina: Layer Segmentation of Retina in OCT Images Using Deep Learning. *Transl Vis Sci Technol.* 2020; 9(2): 61. doi: 10.1167/tvst.9.2.61

[13] Fazekas B, Aresta G, Lachinov D, et al. SD-LayerNet: Semi-supervised Retinal Layer Segmentation in OCT Using Disentangled Representation with Anatomical Priors. In: Wang L, Dou Q, Fletcher PT, Speidel S, Li S. , eds. *Med Image Comput Comput Assist Interv.*Springer Nature Switzerland; 2022; Cham: 320-329

[14] Farshad A, Yeganeh Y, Gehlbach P, Navab N. Y-Net: A Spatiospectral Dual-Encoder Network for Medical Image Segmentation. In: Wang L, Dou Q, Fletcher PT, Speidel S, Li S. , eds. *Med Image Comput Comput Assist Interv.*Springer Nature Switzerland; 2022; Cham: 582-592

[15] Lorenz B, Künzel SH, Preising MN, et al. Single Center Experience with Voretigene Neparvovec Gene Augmentation Therapy in RPE65 Mutation–Associated Inherited Retinal Degeneration in a Clinical Setting. *Ophthalmology* 2023.

[16] Kobler E, Effland A, Kunisch K, Pock T. Total Deep Variation for Linear Inverse Problems. *Proc IEEE Comput Soc Conf Comput Vis Pattern Recognit* 2020: 7546-7555. doi: 10.1109/cvpr42600.2020.00757

[17] Chambolle A. An Algorithm for Total Variation Minimization and Applications. *J Math Imaging Vis* 2004(20). doi: 10.1023/B:JMIV.0000011325.36760.1e
